# Adolescent Participation in HPV Vaccine Clinical Trials: Are Parents Willing?

**DOI:** 10.1007/s10900-017-0331-x

**Published:** 2017-03-21

**Authors:** Jennifer Cunningham Erves, Tilicia L. Mayo-Gamble, Pamela C. Hull, Lauren Duke, Stephania T. Miller

**Affiliations:** 1 Department of Internal Medicine, Meharry Medical College, 1005 Dr. DB Todd Blvd, Nashville, TN 37208-3599 USA; 2 Department of Family and Community Medicine, Meharry Medical College, 1005 Dr. DB Todd Blvd, Nashville, TN 37208 USA; 3 Department of Medicine, Vanderbilt University Medical Center, 2525 West End Ave, Suite 800, Nashville, TN 37203-1738 USA; 4 Lentz Public Health Department, Nashville, TN 37209 USA; 5 Department of Surgery, Meharry Medical College, 1005 Dr. DB Todd Blvd, Nashville, TN 37208 USA

**Keywords:** Adolescents, HPV vaccine, Clinical trials, Parental willingness

## Abstract

Approximately one-quarter of human papillomavirus (HPV) infections are acquired by adolescents, with a higher burden among racial/ethnic minorities. However, racial/ethnic minorities have been underrepresented in previous HPV vaccine trials. Ongoing and future HPV vaccine optimization trials would benefit from racially- and ethnically-diverse sample of adolescent trial participants. This study examined factors influencing parental willingness to consent to their adolescents’ participation in HPV vaccine clinical trials and tested for possible racial differences. A convenience sample of parents of adolescents (N = 256) completed a cross-sectional survey. Chi square analyses were used to assess racial differences in parental HPV vaccine awareness and intentions and willingness to consent to their child participating in an HPV vaccine clinical trial. Ordinal logistic regression was used to identify factors associated with willingness. Approximately 47% of parents were willing to allow their adolescent to participate in HPV vaccine clinical trials (30.7% African American and 48.3% Caucasian, *p* = .081). African Americans had lower HPV vaccine awareness (*p* = .006) but not lower intentions to vaccinate (*p* = .086). Parental willingness was positively associated with the following variables: Child’s age (*p* < .039), Perceived Advantages of HPV Vaccination for Adolescents (*p* = .002), Parental Trust in Medical Researchers (*p* < .001), and Level of Ease in Understanding Clinical Trial Information (*p* = .010). Educating parents about the advantages of HPV vaccines for younger adolescents using low-literacy educational materials and building trust between parents and researchers may increase parental willingness to consent to adolescent participation in HPV vaccine clinical trials.

## Introduction

Cancer incidence associated with the human papillomavirus (HPV) is on the rise [1]. Proliferation of HPV-associated cancers contributes to the already widening gap of racial and ethnic disparities in cancer [1]. HPV has been linked to six types of cancer (cervical, vaginal, vulvar, penile, oropharyngeal, and anal), and incidence of HPV-associated cancers are up to 75% higher for African Americans compared to Caucasians [2]. HPV infection continues to be a public health threat to adolescents [3], especially African American adolescents [4]. Between 2007 and 2010, female adolescents aged 14–19 years accounted for nearly 25% of HPV infections in the U.S. [5]. Although there is currently no HPV test available for male adolescents [6], it is estimated that rates of HPV infection are comparable for males and females [7]. High HPV infection among adolescents makes them an appropriate target for prevention of HPV-related cancers.

The clinical trials that evaluated efficacy of three HPV vaccines (Cervarix, Gardasil-4, and Gardasil-9) for primary prevention against HPV infections were conducted in adolescents and young adults aged 16–26 years [8–11]. Although Cervarix and Gardasil-4 are no longer being marketed in the U.S., the trial findings demonstrated all three vaccines were highly efficacious against the targeted HPV types and associated sequelae. Cervarix and Gardasil-4 vaccines protected against two HPV types (16 and 18) that are responsible for 70 percent of cervical cancers, and Gardasil-9 protects against seven HPV types (16, 18, 31, 33, 45, 52, 58) causing nearly 90 percent of cervical cancers. Preadolescents and adolescents were not recruited to participate in these initial studies that led to vaccine approval due to legal and ethical issues (i.e. sexual activity evaluations and low exposure of HPV) [12]. To address this limitation, subsequent immunogenicity studies were conducted in adolescents aged 9–15 in order to bridge vaccine efficacy findings [12]. In fact, recent evidence of a greater immune response among 9–15 year olds with two doses of Gardasil-9 at least 5 months apart, compared to older ages with three doses, led to revision of the HPV vaccine guidelines in the U.S. in 2016. The current recommendation is for children aged 9–14 years to receive the HPV vaccine in two doses, while those starting the vaccine series at age 15 or older still require three doses [13].

While findings of these international studies demonstrated that the vaccine was efficacious and safe and induced higher immunogenicity under age 15 [12, 14–16], Black participation in these studies was disproportionately low compared to other racial groups [17, 18]. For example, among adolescents aged 9–15 participating in the Gardasil-9 immunobridging clinical trials internationally, only 5.4% were Black compared to 19.2% multi-racial, 13.5% Asian, and 62.0% Caucasian [18]. Prior research suggests that distribution of infection from the various HPV types differs across racial/ethnic groups and global geographic regions [19–21]. Thus, participation of diverse adolescents in future HPV vaccine clinical trials would help ensure the development of new HPV vaccine formulations and guidelines that will optimally benefit all racial/ethnic groups and geographic regions. Emerging preliminary data suggest that one dose of the HPV vaccine could potentially produce a sufficient immune response [22–25]. A clinical trial was conducted in Costa Rica to determine immunogenicity of a single-dose, with plans to conduct a subsequent confirmatory trial in the U.S. [26, 27]. Therefore, there is a need to develop mechanisms to improve participation of racially- and ethnically-diverse adolescents in these future trials.

Parents or legal guardians must give consent for an adolescent child to participate in vaccine clinical trials [28]. The pediatric literature is deficient in identifying factors that influence parents’ willingness to provide consent for their children to enroll in HPV vaccine trials. Our literature searches revealed only one such study by Lazcano-Ponce and colleagues [29] conducted in Mexico, which found that mothers’ understanding of the vaccine and its benefits, along with the mother having had two or more sexual partners, were predictors of adolescent participation in HPV vaccine clinical trials. To our knowledge, a similar study has not been conducted in the U.S.

The purpose of this study was to assess, among parents of African American and Caucasian adolescents ages 9–15 in Tennessee, U.S., factors influencing their willingness to allow their adolescent children to participate in HPV vaccine clinical trials. Using the Health Belief Model (HBM) as an overarching conceptual framework, this study’s objectives were to: (1) determine whether racial differences exist in parental HPV vaccine awareness, HPV vaccine intentions, and their willingness to provide consent for their adolescent’s participation in HPV vaccine clinical trials, and (2) assess the independent association of the various HBM-related factors on parental willingness while controlling for race. Findings from this study will inform strategies to recruit adolescents for upcoming immunogenicity studies and the development of interventions aimed to educate parents about HPV vaccine efficacy studies to inform their decision-making regarding their child’s participation in vaccine clinical trials such as HPV vaccines.

## Methods

### Conceptual Model

We applied the HBM [30] to examine the likelihood that a parent would allow their child to participate in HPV vaccine clinical trials. This model has been successfully applied to past behavioral research [31]. According to key model components, when the anticipated benefits (advantages) of participating in HPV vaccine clinical trials outweigh the perceived barriers (disadvantages), there is an increased likelihood of clinical trial participation [30]. Based on this model, factors that may influence parental willingness include: (1) perceived advantages of adolescents’ participation in clinical trials; (2) perceived disadvantages of adolescents’ participation in clinical trials; and (3) modifying factors (e.g., HPV vaccine awareness, trust in medical researchers, and level of understanding of clinical research information).

### Setting and Sample

From November 2014 to May 2015, we conducted a cross sectional study among a convenience sample of 256 parents across the state of Tennessee. This study (Protocol#: 14-11-306) was approved by the Meharry Medical College Institutional Review Board. Inclusion criteria were: (1) female or male, (2) English-speaking, (3) have, care for, or make medical decisions for an adolescent child aged 9–15 years. If parents/guardians had more than one child in the targeted age range, they were asked to complete the survey in reference to their youngest child in the age range.

Consistent with principles of community-engaged research, we solicited input on recruitment strategies and sources from parents, community members (i.e., clergy, and a variety of other community stakeholders). As a result, parents were recruited in person in a variety of community-based settings (i.e. churches, sorority events, schools, fitness centers, physicians’ offices, and health departments) in Nashville, Tennessee, U.S. We also identified participants in other parts of Tennessee using an online recruitment tool called ResearchMatch.org. ResearchMatch is a national health volunteer registry that allows researchers to identify individuals searching for research studies, and allows individuals to identify research studies of interest [32]. Other online recruitment sources were email and crowdsourcing (i.e., distribution of the survey to a crowd/group of people) [33].

### Data Collection

Both online and in-person respondents first completed informed consent by reviewing the background/purpose of the survey and checking a box online or on paper to indicate their consent to participate in the survey. Participants then completed the anonymous online or paper-based survey. Paper surveys were completed individually or in groups ranging from 2 to 30 participants. Upon completion, per recommendation from the community stakeholders, participants completing the survey in person received a $10 gift card for their time. Online survey respondents filled out a separate compensation form, and a $10 gift card was mailed to them. All study procedures took approximately 15–30 min.

### Data Sources

We combined community-engaged research principles with traditional research study methods to assist in the development of the *HPV Clinical Trial Survey for Parents with Children Aged 9–15* (CTSP-HPV). Steps in the survey development process have been described in detail previously [34]. In brief, in collaboration with the Principal Investigator (JCE), community members, including parents, clergy, and a variety of other community stakeholders provided input on survey content, length, and administration procedures along with recruitment mechanisms [34]. In addition to basic demographic information for parents and adolescents, the CTSP-HPV consisted of seven theoretically-related measures that have been described in detail previously and for which evidence of acceptable reliability and validity have been confirmed and reported [34]. The measures used in the present analysis are described below. [See Cunningham et al. [34] for a full description of the survey development, including reliability and validation procedures].

#### Dependent Variable

*Parental willingness to consent to adolescent participation in a HPV vaccine clinical trial* was measured with one item asking, “How willing would you be to allow your child to participate in a HPV vaccine clinical trial to test the HPV vaccine?” using a 5-point response scale ranging from very unwilling to very willing.

#### Independent Variables

##### Attitudes

*Perceived Benefits (Advantages of HPV Vaccination for Adolescents*) and *Perceived Barriers* (*Disadvantages of HPV Vaccination for Adolescents*). Perceived Benefits was a 4-item measure, and Perceived Barriers was a 5-item measure. For each measure, parents indicated their level of agreement using a 5-point Likert scale. Items related to advantages were represented by statements such as “The HPV vaccine is a good way to protect my child’s health.” “I lack information about the HPV vaccine to decide whether my child should receive it” is an example of a disadvantage-related item. Mean scores were computed for each scale. Higher scores were reflective of more perceived advantages and perceived disadvantages on the respective scales.

##### Modifying Factors

*HPV Vaccine Awareness* Parents were asked, “Have you heard of the Human Papillomavirus (HPV) Vaccine?” with the response options *Yes, No*, and *I don’t know*. Response options *No* and *I don’t know* were combined for the analysis.

*Race* This single item asked parents “How would you describe yourself?” The categorical response options were: (1) *Black, African American, African, or Afro-Caribbean*, (2) *Native American Indian or Alaskan Native*, (3) *Hispanic, Latino, or Spanish origin*, (4) *White*, (5) *Native Hawaiian*/*Other Pacific Islander*, (6) *Middle Eastern*/*North African*, (7) *Asian*, (8) *Prefer not to answer*, and (9) *Other*. The race variable was transformed to the response options *African American, White*, and *Other*.

*Child’s Age* This single item asked parents “Please select the YOUNGEST of your children in this age range. Answer ALL the questions in this survey specifically about this child”. Responses option were 9, 10, 11, 12, 13, 14, and 15.

*HPV Vaccine Intention* Parents were asked, “Which of the following describes how you feel about getting your child the HPV vaccine?” The four ordinal response options from lowest to highest intentions were *I do not plan to get my child the HPV vaccine, I am unsure about getting my child the HPV vaccine, I plan to get my child the HPV vaccine*, and *My child is already vaccinated against HPV*.

*Parental Trust in Medical Researchers* This 12-item measure included statements such as “Researchers who make mistakes try to cover them up.” Response options ranged from 1 (strongly disagree*)* to 5 (strongly agree*)*. Negatively worded questions were reverse coded so that higher scores indicated more trust in medical researchers. The mean score was computed for the scale.

*Level of Ease in Understanding Clinical Trial Information* A single item was used as a proxy measure for health literacy, “Has the information you heard about clinical trials for your child been easy to understand?” The response options included *not at all, a little, some, a great deal, completely*, and *I haven’t received anything*. For the analysis, the responses of not at all, a little, and some were combined into one category, and the responses of a great deal and completely were combined together. The response option *I haven’t received anything* was used as the reference category.

### Data Analysis

Data were analyzed using Version 23 of the IBM Statistical Package for the Social Sciences (SPSS). Due to a small sample (N = 17) of the participants reporting Hispanic or other ethnic origins, these individuals were excluded from the current analysis and comparisons were conducted between Caucasians and African Americans, resulting in an analytic sample size of N = 239 participants. Chi square analyses were used to test the bivariate association between race and HPV vaccine awareness, HPV vaccine intentions, and the degree of willingness to allow their adolescent children’s participation in HPV vaccine clinical trials. Multivariate ordinal logistic regression was used to estimate the independent association of each of the HBM-based CTSP-HPV measures on parental willingness while controlling for socio-demographics (race and child’s age), and the other independent variables. We employed an alpha level of 0.05, while tests with marginal significance (alpha of 0.10) were also noted for exploration in future research.

## Results

### Sample Characteristics

Demographic characteristics for the 239 parents in the analytic sample are shown in Table 1. Most participants were African American (62.8%) and female (80.3%). Annual household income had a roughly even spread with 26.9% reporting an income of less than $20,000 and 23.0% reporting an income $80,000 or more. Half of participants (48%) were married and had a bachelors’ degree or higher (50.1%) (See Table 1). Based on parent reports, the adolescent children of reference for the survey were 51.5% female, 60.7% African American, and mostly of very good to excellent health (85.8%). Most adolescents also had health insurance through parents’ employment (53.6%). (Results are not shown).

**Table 1:** Demographic characteristic of participants

Variable	N	Percentage of sample (%)
Race		
White	89	37.2
Black	150	62.8
Gender		
Male	44	18.4
Female	192	80.3
Other/prefer not to say	2	0.8
Education level		
Some HS/GED or HS diploma	60	25.1
Some college	75	31.4
College/post-graduate	104	43.5
Marital status		
Married	115	48.5
Divorced	25	10.5
Separated	14	5.9
Single/never married	73	30.5
Widowed	8	3.3
Other	2	0.8
Annual household income		
<$20,000	63	26.4
$20,001–$40,000	53	22.2
$40,001–$60,000	34	14.2
$60,001–$80,000	29	12.1
>$80,000	55	23.0

### Racial Differences in HPV Vaccine-Related Variables

Table 2 reports parental HPV vaccine awareness, HPV vaccine intentions, and willingness to allow their adolescent children to participate in HPV vaccine clinical trials by race. Among the HBM-related factors, there was a significant difference in awareness of the HPV vaccine by race, with greater awareness among Caucasian parents (χ^2^(1) = 7.613, *p* = .006). There was not a significant racial difference in parental intentions to obtain the HPV vaccine (χ^2^(2) = .767, *p* = .086), with 36.7% of African American and 40.4% of Caucasian reporting that they planned to do so, and 39.3% of African American and 34.8% of Caucasian parents reporting they were unsure of their plans.

**Table 2:** Racial differences in HPV vaccine-related variables

	African American	%	Caucasian	%	*p* value
HPV vaccine awareness					0.006
Yes	113	75.3	80	89.9	
No	37	24.7	9	10.1	
HPV vaccine intentions					0.086
My child is already vaccinated	22	14.7	12	13.5	
Plan to get the vaccine	55	36.7	36	40.4	
Do NOT plan to get the vaccine	14	9.3	10	11.2	
I am unsure	59	39.3	31	34.8	
Parental willingness of adolescent participation in HPV clinical trial					0.081
Very willing	18	12.0	18	20.2	
Somewhat willing	28	18.7	25	28.1	
Not sure	58	38.7	22	24.7	
Somewhat unwilling	18	12.0	10	11.2	
Very unwilling	28	18.7	14	15.7	

Only 30.7% of African American parents and 48.3% of Caucasian parents stated they would be willing to allow their child to participate in a HPV vaccine clinical trial. While African American parents appeared to be less willing to allow their children to participate in a HPV vaccine clinical trial in comparison to Caucasian parents, the racial difference for this variable was only marginally significant (χ^2^(3) = 8.293, *p* = .081).

### Factors Associated with Parental Willingness

Table 3 lists the results of the ordinal logistic regression analysis used to estimate the adjusted associations of socio-demographic factors and the HBM-related factors on parental willingness to allow their child to participate in an HPV vaccine clinical trial. For socio-demographic factors, *child’s age* was significantly associated with parental willingness while race had marginal significance (*p* = .067). Regarding *child’s age*, for each additional year in age, the odds of a one unit increase in parental willingness were 19% greater (*OR* = 1.19, *p* < .039). For each one unit increase on the *Perceived Advantages of HPV Vaccination for Adolescents* and the *Parental Trust in Medical Researchers* scales (i.e., greater perceived advantages and trust), the odds of each unit increase in parental willingness were 25% and 335% greater (*OR* = 1.25, *p* = .002; and *OR* = 4.35, *p* < .001, respectively). In addition, *Level of Ease Understanding Clinical Trial Information* was positively associated with willingness; specifically, parents with low levels of understanding of clinical trial information prior to the current study (i.e., lower health literacy related to clinical trials) were 69% less willing to allow a child to participate in an HPV vaccine clinical trial compared to parents who had high levels of understanding information (*OR* = 0.31, *p* = .010). *HPV Vaccine Intentions and Perceived Disadvantages of HPV Vaccination for Adolescents* were not found to be significantly associated with willingness while controlling for the other variables in the model.

**Table 3:** Ordinal logistic regression of associations with parental willingness of adolescent participation in HPV vaccine clinical trials

Variable	*OR*	*SE*	CI	*p* value
Socio-demographics				
Race				
African American	2.09	0.84	[0.95, 4.58]	0.067
Caucasian	Ref			
Child’s Age	1.19	0.100	[1.01, 1.40]	0.039
Attitudes				
Perceived advantages	1.94	0.41	[1.27, 2.94]	0.002
Perceived disadvantages	1.25	0.31	[0.76, 2.04]	0.380
Modifying factors				
Trust in medical researchers	4.35	1.57	[2.15, 8.83]	<0.001
HPV vaccine intentions	0.81	0.13	[0.60, 1.10]	0.186
Level of ease understanding clinical trial information				
Haven’t heard any info	0.49	0.22	[0.20, 1.16]	0.103
None/a little/some	0.31	0.14	[0.12, 0.75]	0.010
Mostly/completely	Ref			

## Discussion

The HPV vaccine demonstrates great promise to reduce the prevalence of HPV infection in adolescents and future HPV-related cancer incidence among men and women. Adolescents, especially those of populations disproportionately affected by HPV-related cancers (e.g., African Americans), could greatly benefit from future versions of vaccine that cover a wider range of high-risk HPV types and from further changes in immunization guidelines to reduce the number of doses, given the low rates of compliance observed under the three-dose schedule [35, 36]. To our knowledge, this is the first study in the U.S. to assess whether racial differences exist in parental willingness of adolescent participation in HPV vaccine clinical trials and to examine factors influencing parental willingness of adolescent participation in HPV vaccine clinical trials.

In this study, most of the respondents were aware of the HPV vaccine, and many had intentions to obtain the vaccination. Yet, similar to past studies [37], African Americans were significantly less likely to be aware of the vaccine than Caucasian parents. This could potentially reflect that African Americans are less likely to have received a physician’s recommendation for the HPV vaccine [38]. As it relates to their adolescent participation in HPV vaccine clinical trials, approximately one-third were willing to allow their adolescent to participate. In contract to past studies examining general participation in medical research [39], racial differences in willingness to allow adolescent participation in HPV vaccine clinical trials were not significant, suggesting that parents of both races had similar levels of willingness. However, the marginal significance suggests a need for further research among parents in other cities and states.

This study identified several factors influencing willingness of parents to allow their adolescent to participate in a HPV vaccine clinical trial. Similar to past studies reporting factors influencing adolescent participation in clinical trials in general [40], (*Perceived Advantages of HPV vaccination of Adolescents, Parental Trust in Medical Researchers, Child’s Age, and Level of Ease Understanding Clinical Trial Information*) were identified as positive correlates. The total number of perceived advantages, not surprisingly, along with greater degree of trust in medical researchers, were associated with greater willingness. This finding reflects a need for further research to determine strategies to increase awareness of benefits associated with HPV clinical trial participation among parents of adolescents. The finding that trust was positively associated with willingness suggests a need to reduce misconceptions or skepticism some may perceive about research [40]. Hence, mechanisms should also be identified to build trust between parents and researchers to potentially address the lack of willingness of parents to allow adolescent participation in HPV vaccine clinical research. An important factor associated with parental willingness was ease in understanding trial information compared to those who had difficulty understanding information received regarding trial participation. This finding suggests that improving information regarding clinical trials by meeting lower health literacy levels could potentially improve HPV vaccine clinical trial participation. Last, the finding of parents being more apt to allow their older child to participate versus a younger child is similar to finding of other studies that parents tend to be more likely to vaccinate older adolescent children against HPV than younger children [41]. This could represent a potential barrier to recruiting younger children to participate in immunogenicity studies that seek to include participants as young as age 9 to examine the possibility of lowering the number of doses [35].

### Limitations and Strengths

This study was not without limitations. Survey participants were recruited using a convenience sample. Therefore, findings may not be generalizable to a specific population, and selection bias may have been introduced in the sampling. Because the data were self-reported, there was potential for social desirability bias as participants may have provided answers they perceived favorable to the researchers. There was also potential for recall-bias as participants attempted to recall information related to their awareness of the HPV vaccine and clinical trials. Since this was a cross-sectional study, the determination of temporal sequence and causation was not possible. Last, there may be other factors that influence parental willingness among African Americans and Caucasians differently that were not included in the current model. Despite these limitations, there are some strengths. Community engagement was applied at all phases of the research project which was vital to the success of this project. Furthermore, this study clearly documents the procedures so that the validity of the findings can be assessed by other researchers. Last, this study provides implications for future studies as it relates to recruitment of adolescents in clinical trials involving vaccines.

## Conclusions

Perceived benefits of adolescent clinical trial participation, ease of understanding clinical trials, and trust in medical researchers were positively associated with greater parental willingness of adolescent participation. Since this is among the first study of its kind, further studies should be conducted to assess potential differences between racial groups on willingness to allow a child to participate in an HPV vaccine clinical trial. Future research should also examine the information parents need in order to make an informed decision along with the optimal sources to distribute this information. Based on this study’s findings, one area to advise parents on is the benefits of adolescent clinical trials with an emphasis on the HPV vaccine. The incorporation of community-engaged research approaches may also be beneficial for future studies to develop interventions that address factors to increase parental willingness to participate in HPV vaccine clinical trials.
